# Modulating Mitochondrial Dynamics Mitigates Cognitive Impairment in Rats with Myocardial Infarction

**DOI:** 10.2174/1570159X22666240131114913

**Published:** 2024-01-31

**Authors:** Kewarin Jinawong, Chanon Piamsiri, Nattayaporn Apaijai, Chayodom Maneechote, Busarin Arunsak, Wichwara Nawara, Chanisa Thonusin, Hiranya Pintana, Nipon Chattipakorn, Siriporn C. Chattipakorn

**Affiliations:** 1 Neurophysiology Unit, Cardiac Electrophysiology Research and Training Center, Faculty of Medicine, Chiang Mai University, Chiang Mai 50200, Thailand;; 2 Center of Excellence in Cardiac Electrophysiology Research, Chiang Mai University, Chiang Mai 50200, Thailand;; 3 Cardiac Electrophysiology Unit, Department of Physiology, Faculty of Medicine, Chiang Mai University, Chiang Mai 50200, Thailand;; 4 Department of Oral Biology and Diagnostic Sciences, Faculty of Dentistry, Chiang Mai University, Chiang Mai 50200, Thailand

**Keywords:** Myocardial infarction, cognitive impairment, brain metabolism, mitochondrial fission, mitochondrial fusion, mitochondrial dysfunction

## Abstract

**Background:**

We have previously demonstrated that oxidative stress and brain mitochondrial dysfunction are key mediators of brain pathology during myocardial infarction (MI).

**Objective:**

To investigate the beneficial effects of mitochondrial dynamic modulators, including mitochondrial fission inhibitor (Mdivi-1) and mitochondrial fusion promotor (M1), on cognitive function and molecular signaling in the brain of MI rats in comparison with the effect of enalapril.

**Methods:**

Male rats were assigned to either sham or MI operation. In the MI group, rats with an ejection Fraction less than 50% were included, and then they received one of the following treatments for 5 weeks: vehicle, enalapril, Mdivi-1, or M1. Cognitive function was tested, and the brains were used for molecular study.

**Results:**

MI rats exhibited cardiac dysfunction with systemic oxidative stress. Cognitive impairment was found in MI rats, along with dendritic spine loss, blood-brain barrier (BBB) breakdown, brain mitochondrial dysfunction, and decreased mitochondrial and increased glycolysis metabolism, without the alteration of APP, BACE-1, Tau and p-Tau proteins. Treatment with Mdivi-1, M1, and enalapril equally improved cognitive function in MI rats. All treatments decreased dendritic spine loss, brain mitochondrial oxidative stress, and restored mitochondrial metabolism. Brain mitochondrial fusion was recovered only in the Mdivi-1-treated group.

**Conclusion:**

Mitochondrial dynamics modulators improved cognitive function in MI rats through a reduction of systemic oxidative stress and brain mitochondrial dysfunction and the enhancement of mitochondrial metabolism. In addition, this mitochondrial fission inhibitor increased mitochondrial fusion in MI rats.

## 1 INTRODUCTION

Ischemic heart disease (IHD) is recognized as being a leading cause of death worldwide. An updated report showed that approximately 200 million people were living with IHD in 2019 across many countries [1]. Lacking blood flow to the heart is a major cause of IHD, resulting in myocardial injury and cardiac dysfunction, which is also designated myocardial infarction (MI) [2]. Our previous study has shown that increased systemic oxidative stress was a link between cardiac complications and brain pathologies, resulting in cognitive decline in MI rats [3].

Systemic oxidative stress impaired blood-brain barrier (BBB) integrity, allowing extravasation molecules and free radicals into the brain, leading to a burst of mitochondrial reactive oxygen species (ROS) generation in the brain [4]. ROS are key pathological mediators that cause brain damage in various models, including cardiac ischemia/reperfusion (I/R) injury, prediabetes, and Alzheimer’s disease (AD) [5-8]. A previous study reported that brain metabolism is impaired when ROS production exceeds the threshold levels' limit [9].

Furthermore, excessive ROS induces an imbalance between mitochondrial fission and fusion, which are the mitochondrial quality control processes. The impairment of this mitochondrial quality control process is associated with neurological disorders and neurodegenerative diseases such as AD and Parkinson’s disease [10]. Our previous study demonstrated that the fusion process of mitochondria was reduced in the brain following cardiac I/R injury, which was reversed by mitochondrial fusion promoter (M1) administration, whereas the mitochondria fission process was not affected in this model [11]. Mitochondrial division inhibitor 1 (Mdivi-1) is a mitochondrial fission inhibitor through suppresses Drp1-dependent fission [12]. The beneficial effects of Mdivi-1 on neuroprotection were previously reported following evidence that it suppressed mitochondrial fission, improved brain homeostasis, attenuated brain oxidative stress and preserved synaptic integrity, leading to restoration of cognitive function in rats with doxorubicin-induced chemobrain [13]. However, the effects of the modulation of mitochondrial dynamics on brain function in the case of MI have not been determined.

In the physiological condition, the synaptic function in the brain relies on the metabolizing of glucose by glycolysis and/or mitochondrial oxidative phosphorylation (OXPHOS) [14]. However, glucose is primarily used as the main substrate for generating energy in the brain in the form of ATP *via* glycolysis [15]. A previous study has shown that cognitive impairment is directly correlated with reduced glucose metabolism, particularly in the AD model [16]. Not only glucose but also ketone bodies are utilized in the production of ATP in the brain. However, ketone bodies are considered a minor source of ATP for the brain since this source of energy is only used during oxygen or glucose deprivation [17]. The changes in the brain metabolome involving the brain metabolism following MI and MI with mitochondrial dynamics modulators have never been investigated.

Enalapril is an angiotensin-converting enzyme (ACE) inhibitor, which is strongly recommended as first-choice therapy in patients with heart failure [18]. Enalapril has been selected as a positive control in this study, a decision made following the positive results in our previous studies. That study found that chronic treatment with enalapril effectively reduced cardiac remodeling in MI rats [9, 19]. It has also been shown to effectively reduce brain inflammation in MI mice [20].

All previous findings have informed the objective of the present study, which is to investigate the effects of Mdivi-1 and M1 on cognition and brain pathology, including the levels of AD proteins, oxidative stress, mitochondrial dysfunction, and brain metabolomes, and these parameters are the primary endpoints of this study. The study is carried out on MI rats, with comparisons being made to the effect of enalapril. The hypothesis is that both mitochondrial dynamics modulators improve cognitive function in MI rats through a reduction in oxidative stress and the restoration of brain mitochondrial function and brain metabolism.

## 2 MATERIALS AND METHODS

### 2.1 Animal Preparation

All rats were obtained from the Nomura Siam International Co., Ltd., Bangkok, Thailand. Animals were acclimated for 1 week, under controlled temperature at 21±1°C with a 12:12 hour light and dark rhythm.

### 2.2 Experimental Protocol

Eighty-two male Wistar rats (250-300 g) were anesthetized with isoflurane (Piramal Critical Care, Bethlehem, PA, USA). The rats were intubated with an endotracheal tube and ventilated with a rodent ventilator (CWE. Inc., Ardmore, PA, USA). Left thoracotomy was performed, and MI was induced by the permanent left anterior descending artery (LAD) ligation (n=70). The sham group underwent the left thoracotomy without LAD ligation (n=12). The operation was performed under sterile conditions [9]. After the surgery, Broad-spectrum antibiotics and analgesics were administered to the rats for 7 days, and non-absorbable sutures were removed. The cardiac function was evaluated with transthoracic echocardiography. Surviving rats with a %LVEF < 50 were included in the experimental study (n=52).

After 1-week post-MI, rats were randomly assigned in to 4 sub-groups for receiving the intervention, including the vehicle group; rats were treated with 3% DMSO *via* intraperitoneal injection (IP) as a vehicle; MI+Enalapril (10 mg/kg/day) was administered *via* oral gavage; MI+Mdivi-1 group, 1.2 mg/kg/day of Mdivi-1 (MedChemExpress LLC, USA) and MI+M1 group, 2 mg/kg/day of M1 (Sigma-Aldrich Co., USA). Mdivi-1 and M1 were administered to the MI rats *via* IP once a day for 4 weeks. Enalapril is used as a positive control in this study due to our previous study showed that treatment with 10 mg/kg of enalapril effectively reduced cardiac remodeling in MI rats [9]. The open field test (OFT) and novel object location (NOL) test were used to measure the cognitive function of all rats. The blood sample was collected from the tail tip. Then, the rats were rapidly decapitated, and the brain was collected to determine brain mitochondrial ROS, brain mitochondrial dynamics proteins, hippocampal dendritic spine density proteins, BBB proteins, AD proteins and brain metabolomes. The study protocol is illustrated in Fig. (**1**).

### 2.3 MI Procedure

Permanent left anterior descending artery (LAD) ligation was used to induce MI as described previously [3]. Rats were peritoneally injected with Xylazine (5 mg/kg) and Zoletil (50 mg/kg) to induce anesthesia, where the 50% lethal dose (LD50) for each agent is 121-240 mg/kg [21, 22] and 160 mg/kg [23], respectively. Then, the MI was induced by permanent left anterior descending coronary (LAD) ligation. A left thoracotomy was performed by opening the fourth intercostal space to disclose the heart, and then LAD was permanently ligated. The electrocardiogram (ECG) was monitored to confirm the infarction of the myocardium by S-T segment elevation. At 1-week post-MI, the impairment of cardiac function was confirmed with echocardiography. During this procedure, the rats received 3% isoflurane (Attane, Piramal Critical Care, Bethlehem, USA) and oxygen. S-12 probe was positioned on the chest of the rats at the supine position, followed by M-mode echocardiography and %LVEF was automatically analyzed (Phillips Medical Systems, Massachusetts, USA).

### 2.4 Novel Object Location (NOL) Test

The circular-based box was used as the field test. The first day of the behavioral test was an open field test (OFT). Rats were placed in the middle of the field for 10 minutes to explore the apparatus. Then, on the second day, the familiarization phase was performed. Rats were left in the box to explore the 2 identical objects for 10 minutes. The testing phase was performed on day 3, one of the identical objects was moved to the opposite location, and rats were left in the box to explore the objects for 10 minutes. The time spent exploring each object was calculated as a percent preference index [24].

### 2.5 Serum Oxidative Stress

Systemic oxidative stress was determined by serum malondialdehyde (MDA) levels. The absorbance of thiobarbituric acid reactive substances from a high-performance liquid chromatography (HPLC) method was represented as the levels of serum MDA as previously described [25].

### 2.6 Brain Mitochondrial Reactive Oxygen Species (ROS) Level Measurement

After sacrifice, brain tissue was rapidly removed, and brain mitochondria were extracted as previously described [25]. Hydrogen peroxide (H_2_O_2_) (2 mM) was directly added to the mitochondria to induce a severe oxidative stress condition. The DCF fluorescence intensity was measured by a fluorescent microplate reader (BioTek, Winooski, VT, USA). The levels of brain mitochondrial ROS were represented as the changes in the percentage of DCF fluorescence intensity between mitochondria with and without H_2_O_2_ [3].

### 2.7 Dendritic Spine Staining

The brain slices (400 nm-thickness) were fixed with 4% paraformaldehyde for 1 hour and stained with 1,1′-dioctadecyl-3,3,3′,3’-Tetramethylindocarbocyanine Perchlorate (DiI; Invitrogen, Waltham, MA, USA) for 7 days. Then, the dendritic segments were captured at the CA1 region using a confocal microscope (Olympus FLUOVIEW FV3000, Japan). Two to three brain slices/rat were used for dendritic spine analysis [26].

### 2.8 Western Blot Analysis

The extraction protein from the hippocampus (2 mg/ml) was used to investigate the molecular process by western blot analysis. The electrophoretic separation was conducted through SDS-polyacrylamide gel, and then proteins were transferred to the membrane in the Wet/Tank western blot system (Bio-Rad, USA). Then, membranes were blocked and incubated with primary and secondary antibodies, respectively. Antibodies for immunoblotting were anti-ZO-1 (1:1000 dilution, Santa Cruz, SC-33725), Claudin 5 (1:1000 dilution, Abcam, Ab15106), APP (1:1000 dilution, Cell signaling, 2452), BACE-1 (1:1000 dilution, Abcam, Ab2077), Tau (1:1000 dilution, Cell signaling, 4019), p-Tau (1:1000 dilution, Cell signaling, 12885), SOD2 (1:1000 dilution, Cell signaling, 13194), GPX4 (1:1000 dilution, Abcam, Ab125066), Drp1 (1:1000 dilution, Cell signaling, 5391), phosphorylated-Drp1 (p-Drp1) (1:1000 dilution, Cell signaling, 3455), Mfn1 (1:1000 dilution, Abcam, Ab221661), Mfn2 (1:1000 dilution, Cell signaling, 9482), OPA1 (1:1000 dilution, Cell signaling, 80471), PGC-1α (1:1000 dilution, Abcam, Ab54481), and β-actin (1:1000 dilution, Cell signaling, SC47778). The visualized protein was conducted using a chemidoc touching system (Bio-rad, USA), and protein density was analyzed by an image J program (National Institute of Health, USA).

### 2.9 Brain Metabolomic Analysis

#### 2.9.1 Brain Metabolome Extraction

After sacrifice, the brain was removed and kept at -80°C. For the extraction process, 1 mL of the extraction solvent containing 7:3 of HPLC-grade methanol: ultrapure water was added to 40 mg of the pulverized brain tissue. The samples were sonicated for 15 sec, then centrifuged at 16,800 g for 10 min at 4°C. The supernatant was used for MS analyses. The dried pellet was weighted to normalize metabolome levels in each brain tissue sample.

#### 2.9.2 Mass Spectrometry Analyses for Brain Metabolomics Studies

A 1260 infinity II LC/6546 Q-TOF MS (Agilent Technologies, Santa Clara, CA, USA) under hydrophilic interaction liquid chromatography (HILIC) negative ion mode was used to determine the levels of brain metabolome, including amino acid, Kreb’s cycle metabolite, nucleotide, lactate, acetoacetate, and 2,3-dihydroxybenzoate. The HILIC protocols were described in a prior study [27].

The injection volume for brain tissue samples was 10 µL. For MS parameters, dual Agilent jet stream electrospray ionization (AJS-ESI) were as follows: full-scan MS detection (m/z 50 to 1,200), acquisition rate 1 spectrum/sec, capillary voltage 3,500 V, nozzle voltage 1,500 V, gas temperature 350°C, drying gas 10 L/min, nebulizer pressure 20 psig, and reference mass correction enabled.

#### 2.9.3 Peak Area Normalization for Brain Metabolomes

The peak area of each metabolome was quantified using MassHunter Quantitative Analysis Software version 10.1 (Agilent Technologies, Santa Clara, CA, USA). Then, the quantitated peak areas of metabolomes with external standards (both with and without stable isotope labelled internal standards) were converted to concentrations according to the calibration curve of each metabolome. Then, peak areas and concentrations of metabolomes with no stable isotope labelled internal standards were normalized under a normalization tool for multi-batch metabolomics data (MetaboDrift), as described previously [27]. Lastly, the final concentrations or peak areas of brain metabolomes were normalized by the dried weight of brain tissue.

## 3 EXPERIMENTAL

All data were presented as mean ± SD and data were processed using GraphPad Prism software (version 7, GraphPad Software, Inc., USA). The normality of distribution was tested by Shapiro-Wilk. For multiple group comparisons, one-way ANOVA followed by an LSD post-hoc test was used to compare the means among groups as indicated throughout the manuscript. 95% coefficient intervals were calculated for each comparison. Kruskal-Wallis with Uncorrected Dunn’s test was applied to the data with a non-normal distribution, including ZO-1 and claudin 5 protein expression. A *p <* 0.05 is considered to be statistically significant.

## 4 RESULTS

### 4.1 The Mitochondrial Fission Inhibitor, Mitochondrial Fusion Promoter and Enalapril Equally Attenuated Cardiac Dysfunction and Effectively Decreased Systemic Oxidative Stress in MI Rats

**Fig. (2):**
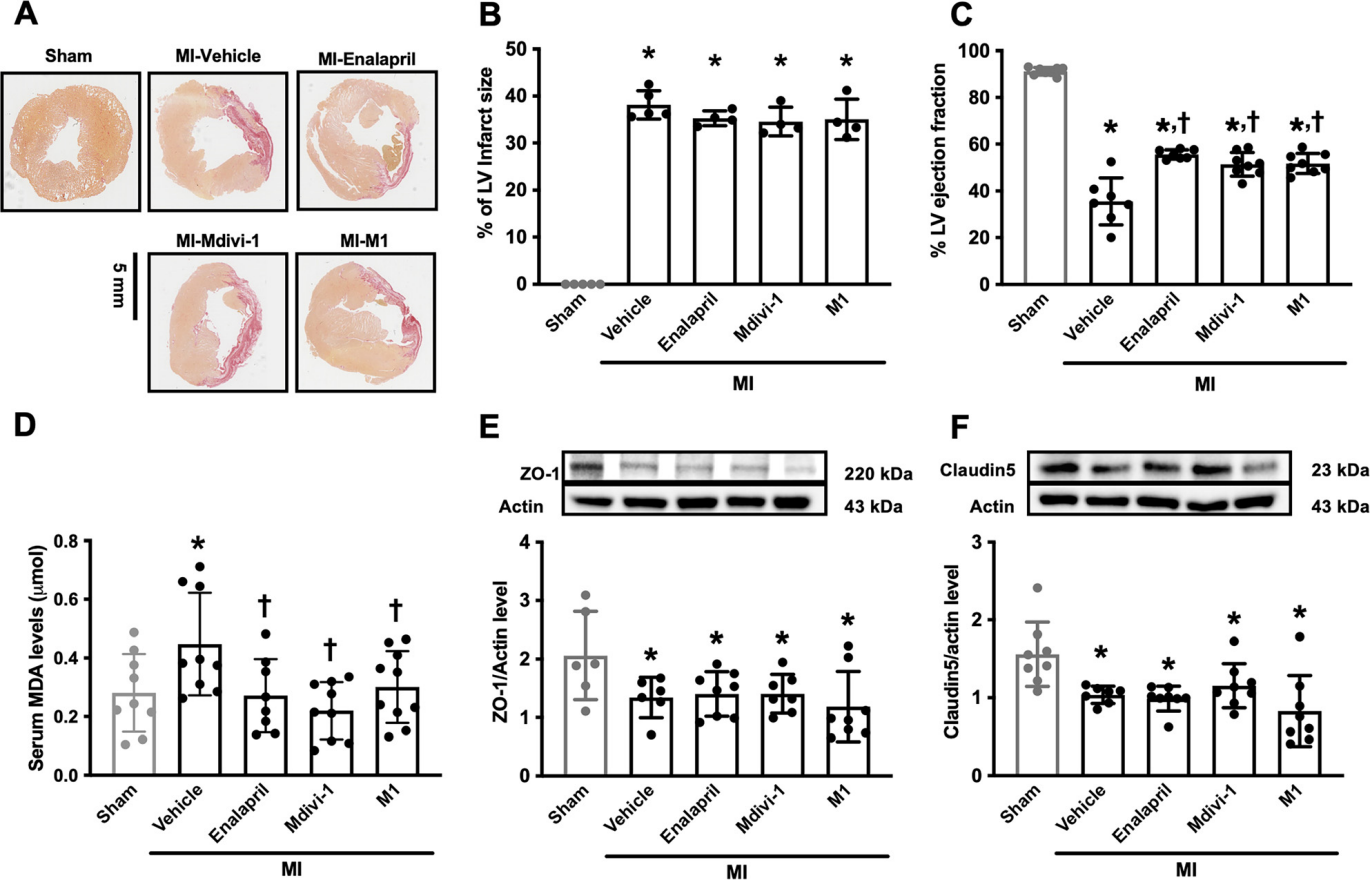
The effects of mitochondrial modulators on systemic oxidative stress and BBB integrity in MI rats. (**A**) the representative of LV infarction detected by Sirius Red staining (the infarct areas were shown in red color), (**B**) % of LV infarct size of LV, (**C**) % LV ejection fraction at 5 weeks-post MI measured by echocardiography, (**D**) Systemic oxidative stress level (n=12 in sham group, n=10 in each of the MI groups), (**E**, **F**) blood-brain barrier protein levels (n=5-8/group). *p < 0.05 *vs*. sham, †*p <* 0.05 *vs*. MI-vehicle. **Abbreviations**: MI: myocardial infarction; LV: left ventricle; MDA: malondialdehyde. The original western blot of blood brain barrier protein expression is shown in Fig. (**S1**).

Myocardial infarction was observed in all MI rats at approximately 35% of the LV area, and the infarct size was not different among MI groups (Figs. **2A**, **B**). Cardiac dysfunction, as indicated by a reduction of left ventricular injection fraction (%LVEF) to less than 50%, was found in MI rats at 1-week post-MI. At 5 weeks post-MI, LVEF was still less than 50% in the vehicle group (91.23 ± 1.629, 35.53 ± 10.10 in sham and MI-vehicle, respectively), which was significantly lower than the sham group (95% CI: 50.00 to 61.39, *p <* 0.05) (Fig. **2C**). %LVEF was significantly increased in all treatment groups, when compared to the vehicle group (95% CI: -26.05 to -14.29, -21.63 to -10.24, -21.98 to -10.59 in MI treated with enalapril, Mdivi-1 and MI, respectively; *p <* 0.05) (Figs. **2C**, **2D**), suggesting that Mdivi-1 and M1 administration attenuated cardiac dysfunction in MI rats to a similar extent as the enalapril treatment.

In MI rats, serum malonaldehyde (MDA) levels were increased in the vehicle group, compared with the sham group (95% CI: -0.2929 to -0.03965; *p <* 0.05) (Fig. **2B**), indicating that systemic oxidative stress had increased in the MI rats. Treatment with Mdivi-1, M1, and enalapril effectively reduced serum MDA levels in comparison to the vehicle group (95% CI: 0.1008 to 0.3540, 0.02291 to 0.2697, 0.04546 to 0.3065, in MI treated with Mdivi-1, MI and enalapril, respectively; *p <* 0.05) (Fig. **2B**). These data indicated that Mdivi-1 and M1 reduced systemic oxidative stress in MI rats to a similar extent as the enalapril treatment.

### 4.2 Enalapril and Mitochondrial Modulators did not Protect BBB Breakdown in MI Rats

In the MI rats, BBB breakdown was observed by a reduction in ZO-1 and claudin 5 protein levels in all MI groups, compared with the sham group (95% CI of ZO-1: 0.1233 to 1.314, 0.09999 to 1.214, 0.07986 to 1.227, 0.3165 to 1.430; 95% CI of claudin5: 0.2001 to 0.8437, 0.2594 to 0.8812, 0.1638 to 0.8074, 0.4184 to 1.040 in Sham *vs* MI treated with vehicle, enalapril, Mdivi-1 and M1, respectively; *p <* 0.05). Our results demonstrated that all treatments did not alter ZO-1 and claudin-5 protein levels in MI rats, compared with the vehicle group (Figs. **2E**, **F**).

### 4.3 The Mitochondrial Fission Inhibitor, Mitochondrial Fusion Promotor, and Enalapril Equally Improved Cognitive Function in MI Rats

**Fig. (3):**
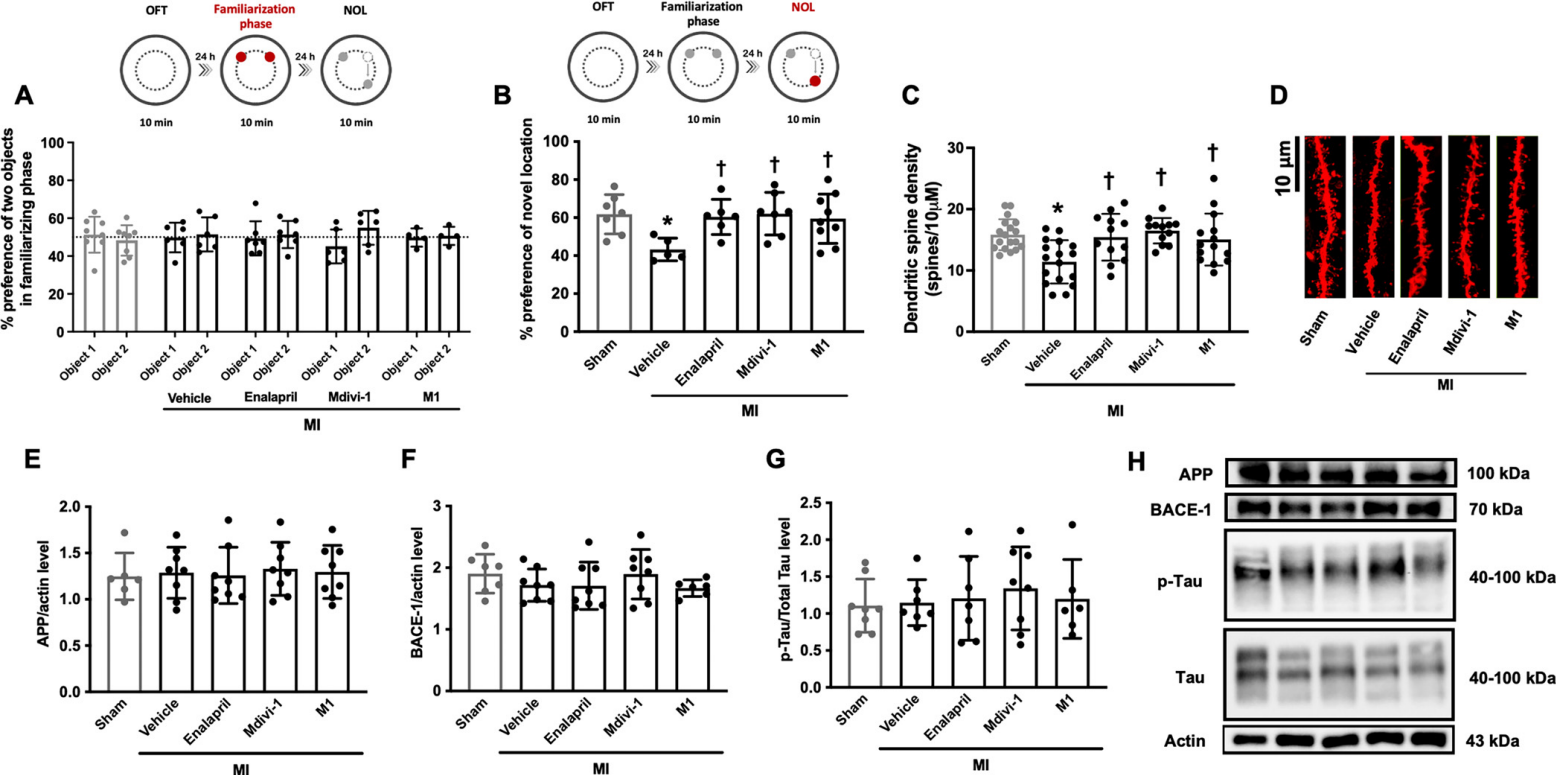
The effects of mitochondrial modulators on cognitive function and APP, BACE-1, Tau and p-Tau proteins in the brains of MI rats (n=7-10 in the sham group, n=6-8 in each of the MI group). (**A**) % time preference of the object in the familiarization phase of NOL test, (**B**) % time preference of the object in the testing phase of NOL test, (**C**) the levels of dendritic spine density in hippocampal CA1, (**D**) the representative of dendritic spine density in hippocampal CA1, (**E**-**G**) the levels of expression of APP, BACE-1, Tau and p-Tau proteins in the hippocampus, (**H**) the representative of APP, BACE-1, Tau and p-Tau proteins expression from WB. *p < 0.05 *vs*. sham, †*p <* 0.05 *vs*. MI-vehicle. **Abbreviations**: MI: myocardial infarction; OFT: open filed test; NOL: novel object location test; APP: amyloid precursor protein; BACE-1: Beta-secretase 1. The original western blot of APP, Bace-1, Tau and p-Tau protein protein expression is shown in Fig. (**S2**).

Our data demonstrated that MI rats had cognitive impairment. The data from the novel object location (NOL) test showed that preference for novel location decreased significantly in the vehicle group, compared with the sham group (95% CI: 5.781 to 31.39; *p <* 0.05), while preference for two objects in the familiarization phase did not differ between groups (Figs. **3A**, **B**). All treatments significantly increased the preference of the novel location, compared with the vehicle group (95% CI: -30.37 to -3.887, -31.67 to -6.055, -28.40 to -4.004, in MI treated with enalapril, Mdivi-1 and M1, respectively; *p <* 0.05) (Figs. **3A**, **B**), suggesting that Mdivi-1, M1, and enalapril improved cognitive function to an equal extent in MI rats.

Consistent with the cognitive function, dendritic spine density was reduced in vehicle group, compared with the sham group (95% CI: 2.180 to 6.663, *p <* 0.05) (Figs. **3C**, **D**), and all treatments increased dendritic spine density in MI rats, compared with the vehicle group (95% CI: -6.511 to -1.513, -7.568 to -2.570, -6.026 to -1.242 in MI treated with enalapril, Mdivi-1 and M1, respectively; *p <* 0.05) (Figs. **3C**, **D**). Furthermore, APP, BACE-1, Tau and p-Tau protein levels were investigated in this study. Our data showed that these protein levels did not differ among groups (Figs. **3E-H**). These results suggested that cognitive impairment at 5 weeks post-MI did not depend on the expression of APP, BACE-1, Tau and p-Tau.

### 4.4 The Mitochondrial Fission Inhibitor, Mitochondrial Fusion Promoter and Enalapril Effectively Attenuated Brain Mitochondrial Dysfunction in MI Rats

**Fig. (4):**
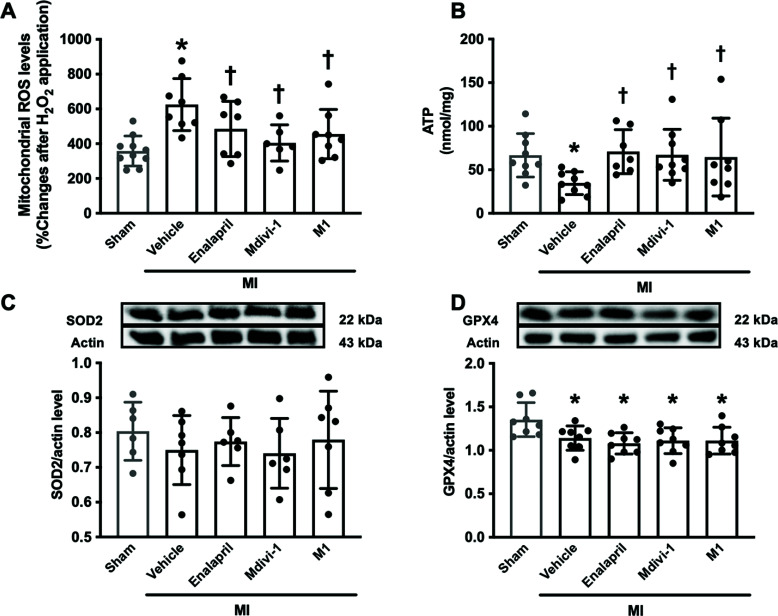
The effects of mitochondrial modulators on oxidative status and total ATP production in the brain of MI rats (n=8-10 in the sham group, n=6-8 in each MI group). (**A**) the level of brain mitochondrial ROS, (**B**) the level of brain ATP, and (**C**, **D**) the levels of antioxidant protein expression. *p < 0.05 *vs*. sham, †*p <* 0.05 *vs*. MI-vehicle. **Abbreviations**: MI: myocardial infarction; ROS: reactive oxygen species; ATP: adenosine triphosphate; SOD2: superoxide dismutase 2; GPX4: glutathione peroxidase 4. The original western blot of antioxidant protein expression is shown in Fig. (**S3**).

Brain mitochondrial function was assessed in this study as an indicator of an energy production source. In MI rats, mitochondrial ROS levels were increased (95% CI: -391.5 to -141.8; *p <* 0.05), and ATP levels were decreased (95% CI: 4.413 to 59.65; *p <* 0.05) in the vehicle group, compared with the sham group (Figs. **4A**, **B**). All treatments significantly reduced mitochondrial ROS levels (95% CI: 3.778 to 276.2, 78.23 to 362.5, 37.82 to 301.0 in MI treated with enalapril, Mdivi-1 and M1, respectively; *p <* 0.05) and increased ATP levels (95% CI: -65.72 to -6.669, -60.05 to -4.814, -58.37 to -1.432 in MI treated with enalapril, Mdivi-1 and M1, respectively; *p <* 0.05), compared with the vehicle group (Figs. **4A**, **B**). In addition, the level of antioxidants was investigated using SOD2 and GPX4 protein levels. Our results showed that SOD2 levels did not differ between groups (Fig. **4C**), whereas GPX4 levels were reduced in all groups of MI rats, compared with the sham group (95% CI: 0.05520 to 0.3682, 0.1168 to 0.4298, 0.08510 to 0.3981, 0.08492 to 0.3979 in MI treated with vehicle, enalapril, Mdivi-1 and M1, respectively; *p <* 0.05) (Fig. **4D**). These data indicated that Mdivi-1, M1, and enalapril equally reduced brain mitochondrial ROS and promoted ATP production in MI rat, without the antioxidant effects.

**Fig. (5):**
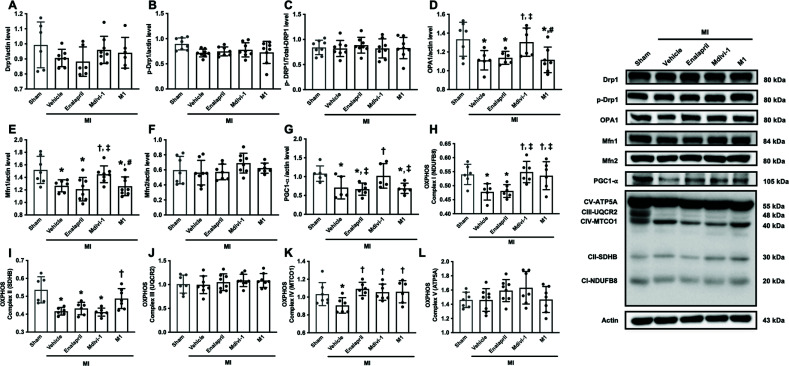
The effects of mitochondrial modulators on brain mitochondrial dynamics and oxidative phosphorylation in MI rats (n=6-8 in the sham group, n=6-8 in each MI group). (**A**-**C**) the levels of brain mitochondrial fission proteins, (**D**-**F**) the level of mitochondrial dynamic proteins, (**G**) the level of mitochondrial biogenesis protein, (**H**-**L**) the levels of OXPHOS complex I-V. *p < 0.05 *vs*. sham, †*p <* 0.05 *vs*. MI-vehicle, ‡*p <* 0.05 *vs*. MI-Enalapril, #*p <* 0.05 *vs.* MI-Mdivi-1. **Abbreviations**: MI: myocardial infarction; Drp1: dynamin-related protein; p-Drp1: phosphorylated-Drp1; OPA1: optic atrophy 1, Mfn1: mitofusin 1, Mfn2: mitofusin 2, PGC-1α: peroxisome proliferator-activated receptor gamma coactivator-1 alpha, OXPHOS: oxidative phosphorylation.

We also investigated mitochondrial dynamics proteins, including those pertaining to mitochondrial fission (p-Drp1^ser616^ and Drp1) and mitochondrial fusion (OPA1, Mfn1, and Mfn2). Our results showed that levels of Drp1, p-Drp1^ser616^, and p-Drp1^ser616^/Drp1 proteins did not differ between groups (Figs. **5A**-**C**), suggesting that brain mitochondrial fission did not occur in the MI rats. In addition, OPA1 and Mfn1 levels in the MI-vehicle group were lower than the sham group (95% CI; 0.06883 to 0.3786, *p <* 0.05; 0.06812 to 0.4387, *p <* 0.05, respectively) (Figs. **5D**, **E**). Only treatment with Mdivi-1 increased the levels of OPA1 and Mfn1 (95% CI; -0.3536 to -0.03216, *p <* 0.05; -0.3636 to -0.003884, *p <* 0.05, respectively), while other treatments did not affect OPA1 and Mfn1 levels, in comparison with the vehicle group (Figs. **5D**, **E**). There was no significant difference in Mfn2 levels between the groups (Fig. **5F**). Our findings suggested that only Mdivi-1 promoted mitochondrial fusion in MI rats.

Previous studies suggested that mitochondrial fusion is mediated by PGC-1α [28, 29]. Therefore, the level of PGC-1α, a mitochondrial biogenesis regulator, was measured, along with OXPHOS protein levels. Our data showed that, in MI rats, PGC-1α level was decreased in the vehicle groups, compared with the sham group (95% CI: 0.08253 to 0.6596, *p <* 0.05) (Fig. **5G**). Only treatment with Mdivi-1, but not other treatments, increased PGC-1α levels in MI rats, compared with the vehicle group (95% CI: -0.6125 to -0.009704, *p <* 0.05) (Fig. **5G**). A disruption in mitochondrial OXPHOS proteins was found in MI rats, the levels of OXPHOS complex I, II and IV being decreased in the vehicle group, in comparison with the sham group (95% CI: 0.01661 to 0.1082, *p <* 0.05; 0.06461 to 0.1751, *p <* 0.05; 0.007924 to 0.2451, *p <* 0.05) (Fig. **5H**, **I**, **K**). Mdivi-1 and M1 administration increased OXPHOS complex I levels (95% CI: “-0.1167 to -0.02511, *p <* 0.05; -0.1036 to -0.01198, *p <* 0.05) (Fig. **5H**), while M1 increased OXPHOS complex II levels (95% CI: -0.1242 to -0.01775, *p <* 0.05) (Fig. **5I**), and all treatments increased the levels of complex IV in MI rats, compared with the vehicle group (95% CI: -0.3076 to -0.06147, -0.2668 to -0.02970, -0.2761 to -0.03001 in MI treated with enalapril, Mdivi-1 and M1 respectively, *p <* 0.05) (Fig. **5K**). However, there were no differences in the OXPHOS complex III and IV levels between the groups. (Figs. **5J**, **L**). These data suggested that Mdivi-1 promoted mitochondrial biogenesis and mitochondrial OXPHOS complex levels, while M1 and enalapril increased mitochondrial OXPHOS complex levels without mitochondrial biogenesis alteration.

### 4.5 The Mitochondrial Fission Inhibitor, Mitochondrial Fusion Promoter and Enalapril Equally Promoted Brain Mitochondrial Metabolism in MI Rats

**Fig. (6):**
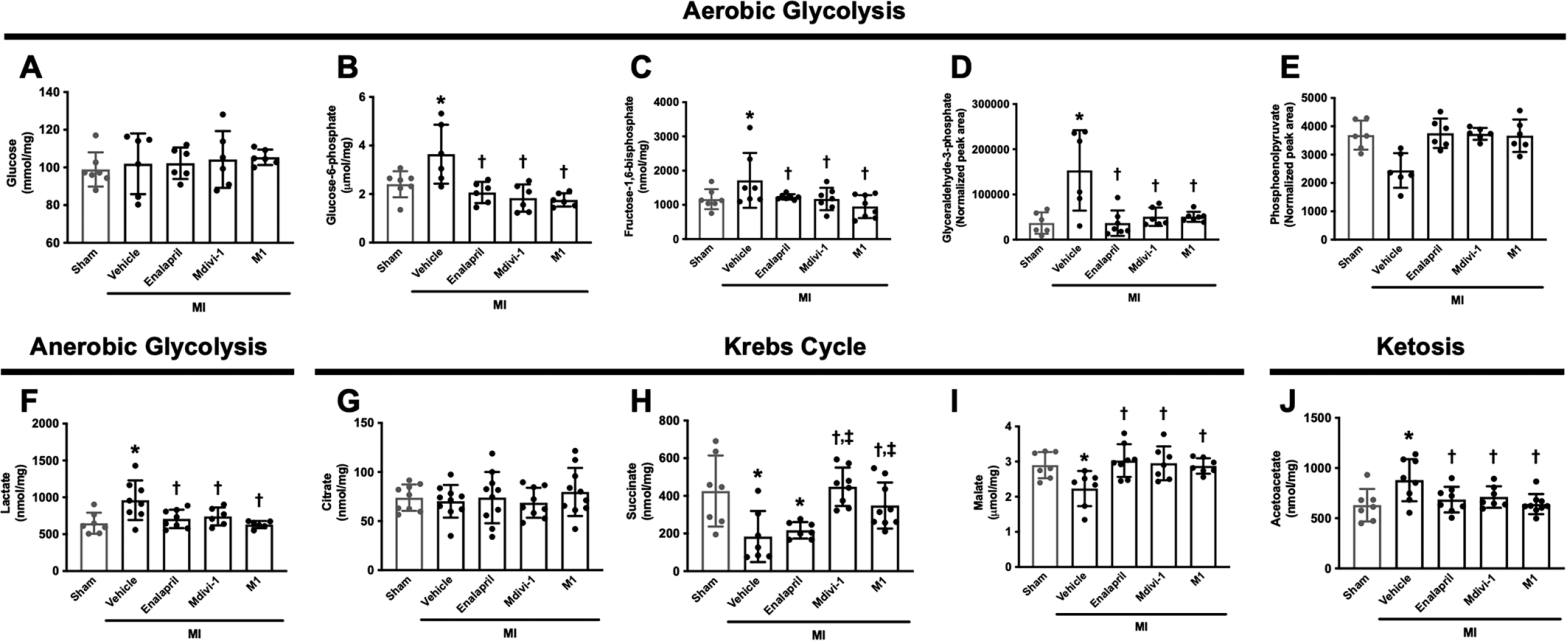
The effects of mitochondrial modulators on brain metabolomic levels of MI rats (n=7-10 in the sham group, n=7-10 in each MI group). (**A**-**E**) the levels of brain glucose and substrates involved in the aerobic glycolysis pathway, (**F**) the level of brain lactate in anaerobic glycolysis, (**G**-**I**) the levels of substrates in Kreb’s cycle, (**J**) the level of acetoacetate in ketosis. *p < 0.05 *vs*. sham, †*p <* 0.05 *vs*. MI-vehicle; ‡*p <* 0.05 *vs*. MI-enalapril. **Abbreviations**: MI: myocardial infarction.

Despite evidence regarding the advantages of mitochondrial dynamics modulators on brain mitochondrial function, metabolomic analysis was carried out to determine energy metabolism in the brain of MI rats. Glucose, glucose-6-phosphate, fructose-1,6-bisphosphate, glyceraldehyde-3-phosphate, and phosphoenolpyruvate were measured as metabolites from the glycolytic pathway (Figs. **6A**-**E**). In MI rats, glucose-6-phosphate, fructose-1,6-bisphosphate, and glyceraldehyde-3-phosphate levels were increased in the vehicle group, in comparison with the sham group (95% CI: -2.014 to -0.4571, -1022 to -76.29, -167255 to -65512 respectively, *p <* 0.05) (Figs. **6B**-**D**). All treatments significantly reduced these glycolysis metabolite levels in comparison with the vehicle group (Figs. **6B**-**D**). However, glucose and Phosphoenolpyruvate levels did not differ between groups (Figs. **6A**, **E**). Lactate was used as a representative of the anaerobic glycolytic pathway, and these were increased in MI rats treated with the vehicle when compared with the sham group (95% CI: -487.5 to -137.6, *p <* 0.05) (Fig. **6F**). All treatments effectively reduced lactate levels in MI rats (95% CI: 85.37 to 423.4, 38.26 to 403.3, 147.5 to 512.6 in MI treated with enalapril, Mdivi-1 and M1 respectively; *p <* 0.05) (Fig. **6F**). Citrate, succinate, and malate are important metabolites in Krebs’s cycle. We found that succinate and malate levels in the brain of MI rats were decreased in the vehicle group, in comparison with those of the sham group (95% CI; 104.3 to 379.4, *p <* 0.05; 0.2081 to 1.129, *p <* 0.05) (Figs. **6H**, **I**). Mdivi-1 and M1 administration increased succinate levels (95% CI: -397.7 to -131.4 and -294.7 to -35.29, *p <* 0.05), and all treatments enhanced malate levels in MI rats, compared with the vehicle group (95% CI: -1.243 to -0.3514, -1.181 to -0.2595 and -1.104 to -0.1834 respectively, *p <* 0.05) (Figs. **6H**, **I**). Citrate levels did not differ between groups (Fig. **6G**). The ketosis pathway was determined by measurement of acetoacetate levels. In the brain of MI rats, acetoacetate levels were upregulated in the vehicle group, in comparison with the sham group (95% CI: -402.8 to -95.48, *p <* 0.05) (Fig. **6J**). All treatments effectively reduced acetoacetate levels in MI rats, compared with the vehicle group (95% CI: 45.02 to 341.9, 14.02 to 321.3, 94.34 to 382.9 in MI treated with enalapril, Mdivi-1 and M1 respectively, *p <* 0.05) (Fig. **6J**). These results suggested that all treatments promoted mitochondrial metabolism to an equal extent and reduced aerobic glycolysis, anaerobic glycolysis, and ketosis metabolism in MI rats.

## 5 DISCUSSION

The data from this study indicated that MI led to cognitive impairment, which was mediated by systemic and brain oxidative stress, mitochondrial dysfunction, an increase in brain glucose metabolites, and a decrease in brain mitochondrial metabolites. Treatment with mitochondrial dynamics modulators, including Mdivi-1 and M1, reduced cognitive impairment in MI rats to an equal extent as the enalapril treatment. There are several mechanisms involved in these beneficial effects conferred by all treatments, including 1) a reduction in cardiac dysfunction and systemic oxidative stress, 2) increased dendritic spine density, and 3) a reduction in brain mitochondrial dysfunction and the promotion of mitochondrial metabolism.

Cognitive impairment is associated with some patients with heart failure [30]. We have reported previously that cardiac dysfunction, oxidative stress, and brain mitochondrial dysfunction contribute to the development of cognitive impairment in MI rats [3]. Cardiac dysfunction was found in MI rats, as indicated by a drop in %LVEF to less than 50%. A recent study reported that a reduction of %LVEF is associated with a reduced %cerebral blood flow in rats with vascular dementia [31]. Therefore, we speculated that cerebral blood flow was reduced in the MI rats, and inadequate cerebral perfusion was responsible for the cognitive impairment observed in our MI rats. During MI, oxidative stress is generated throughout the body, which affects BBB integrity. A rupture of the BBB was found in the MI rats, which allows the leakage of ROS from the systemic circulation into the brain. In the model of ischemic stroke, activation of mitochondrial permeability transition pores (mPTP) and inner membrane anion channels (IMAC) are the major routes of ROS penetration into the mitochondria, and the ROS-induced ROS-released (RIRR) mechanism is activated [32]. We postulated that RIRR plays a major role in the generation of brain mitochondrial oxidative stress in our MI rats. High cerebral ROS levels cause mitochondrial dynamics imbalance, resulting in neuronal cell death [33]. Although enalapril and mitochondrial dynamics modulators effectively restored systemic and brain oxidative stress, they did not upregulate BBB tight junction proteins. This finding suggested that restoration of BBB requires other mechanisms rather than mitochondrial pathways. Previous studies demonstrated that neovascularization and angiogenesis are important in the reconstruction of the BBB in several diseases [34, 35]. In the MI model, cerebral hypoperfusion potentially disrupts BBB integrity and contributes to cognitive dysfunction [36]. Treatment with enalapril and mitochondrial dynamics modulators attenuated cardiac dysfunction, but it might not be sufficient to improve cerebral perfusion in MI rats. As a result, not all these interventions influenced BBB.

In addition, the pathophysiology of cognitive impairment is associated with a disturbance in bioenergetics, including an increase in mitochondrial oxidative stress and impaired glucose metabolism [37]. Synaptic transmission relies on glucose metabolism, the glucose being metabolized by aerobic glycolysis and/or mitochondrial oxidative phosphorylation [14]. In our study, MI increased aerobic and anaerobic glycolysis ketosis and suppressed mitochondrial oxidative phosphorylation, which led to a reduction in ATP levels. A previous study reported that ATP is one of the factors mandatories for dendritic growth [38]. In an AD model, cognitive impairment was found along with abnormal oxidative phosphorylation, glucose hypometabolism, oxidative phosphorylation, and apoptosis [39]. Our study is the first to demonstrate the alteration in brain metabolites during MI; unlike other brain diseases, glucose metabolites were dramatically increased, whereas mitochondrial oxidative phosphorylation was suppressed. Nevertheless, cognitive impairment is still observed, and an increase in glucose metabolism is suggested as a compensatory mechanism of the brain during MI, with mitochondria, therefore, being the main therapeutic target to reduce cognitive impairment in MI rats.

The beneficial effects of mitochondrial dynamics modulators have been reported in several brain injury models, including those involving chemotherapy-induced brain injury, cardiac damage-induced brain injury, and sepsis-induced brain injury [11, 13, 40, 41]. In our current study, administration of Mdivi-1 and M1 effectively reduced systemic oxidative stress in the MI rats to a similar extent as enalapril, which alleviated the RIRR phenomenon, resulting in a decrease in brain mitochondrial oxidative stress level. As a consequence of its biochemical structure, the compound Mdivi-1 serves as a scavenger of free radicals [42]. There is limited evidence concerning the role of M1 as a scavenger of free radicals [43]. We postulated that it reduced oxidative stress levels, leading to a reduction in systemic oxidative stress. It has been reported that free radicals directly attack OXPHOS complexes [44]. In this study, both Mdivi-1 and M1 reduced mitochondrial oxidative stress, thereby increasing the level of expression of mitochondrial OXPHOS complexes, resulting in an increase in ATP production in the brain of MI rats.

Treatment with Mdivi-1, M1, and enalapril effectively promoted mitochondrial oxidative phosphorylation and reduced glycolysis and ketosis, which could restore the ATP levels and increase dendritic spine density in the MI rats. These molecular observations also supported the observed improvement in cognitive function in our MI rats. Although brain oxidative stress was found in the MI rats, the levels were not high enough to induce mitochondrial fission. However, mitochondrial fusion was suppressed. Mitochondrial fusion is important for the oxidative phosphorylation occurring in the mitochondria to maintain normal cell function; however, suppression would limit ATP production [45]. In our study, Mdivi-1, M1, and enalapril did not affect mitochondrial fission. It is interesting that Mdivi-1 increased mitochondrial fusion, and this effect surpasses its ability to inhibit mitochondrial fission. It has been shown that PGC-1α can promote mitochondrial fusion [46], and our data showed that Mdivi-1upregulated the level of Mfn1 and PGC-1α, resulting in promoted mitochondrial fusion. Additionally, it’s worth noting that PGC-1α activates the expression of mitochondrial transcription factor A (TFAM), which is a mitochondrial DNA (mtDNA) binding protein essential for genome maintenance [47]. Upregulated TFAM transcription is generally concomitant with increased mtDNA, suggesting that TFAM regulates mitochondrial biogenesis [48]. Consistency with a previous study, PGC-1α stimulates its own expression and the expression of mitochondrial biogenesis, including TFAM and dynamic gene, Mfn1, leading to a shift of mitochondrial dynamics towards fusion, renders neuronal cells more resistant to stress stimuli [46]. Taken together, our findings could suggest that Mdivi-1 had an efficacy in promoting mitochondrial fusion and mitochondrial biogenesis. However, the precise mechanisms underlying the association between pharmacological intervention and mitochondrial biogenesis should be further investigated.

Data from clinical studies demonstrated that ACE inhibitors improved cognitive function in patients with heart failure, possibly through an increase in cerebral blood flow [49]. In our study, we showed that enalapril shared similar efficacy to both mitochondrial dynamics modulators in improving cognitive function by reducing oxidative stress, brain mitochondrial dysfunction, and enhancing ATP production, but it did not alter mitochondrial fusion in MI rats. Additionally, mitochondrial dynamics modulators and enalapril improved %LVEF in MI rats, which can imply that both treatments might increase cerebral blood flow and lead to the improvement of cognitive function. A direct measurement of cerebral blood flow was not performed in this study, and this is considered as being a limitation of this study.

In addition to MI, both mdivi-1 and M1 have shown favorable effects in mitigating cardiac ischemia/reperfusion (I/R) injury and the associated brain damage following cardiac I/R injury [50, 51]. Some pharmacological interventions have reportedly served as cognitive enhancers during myocardial infarction (MI), such as antiplatelet therapy, statins, Renin-Angiotensin system (RAS) inhibitors, and beta-blockers [52]. However, the molecular investigation of these drugs has not been investigated. In our study, we performed a head-to-head comparison between mitochondrial dynamics modulator and enalapril, which is one of the RAS inhibitors. Our data showed that both mitochondrial dynamics modulators provided similar benefits to enalapril in improving cognitive function, restoring dendritic spine density, attenuating brain mitochondrial ROS production and providing better efficacy than enalapril in promoting mitochondrial fusion and enhancement of brain metabolism. This study demonstrated that enalapril exerted beneficial effects on the brain in a 5-week post-MI model. However, the longer post-MI condition has not been investigated. It is possible that an adjunctive treatment of enalapril and Mdivi-1 would have greater efficacy in both the heart and brain than monotherapy in that condition.

## CONCLUSION

Mitochondrial fission inhibitor and mitochondrial fusion promotor equally restored cognitive function in MI rats through a reduction of systemic oxidative stress and brain mitochondrial ROS production, along with the enhancement of brain mitochondrial metabolism. In addition, mitochondrial fission inhibitors exerted the ability to increase brain mitochondrial fusion in MI rats.

## Figures and Tables

**Fig. (1):**
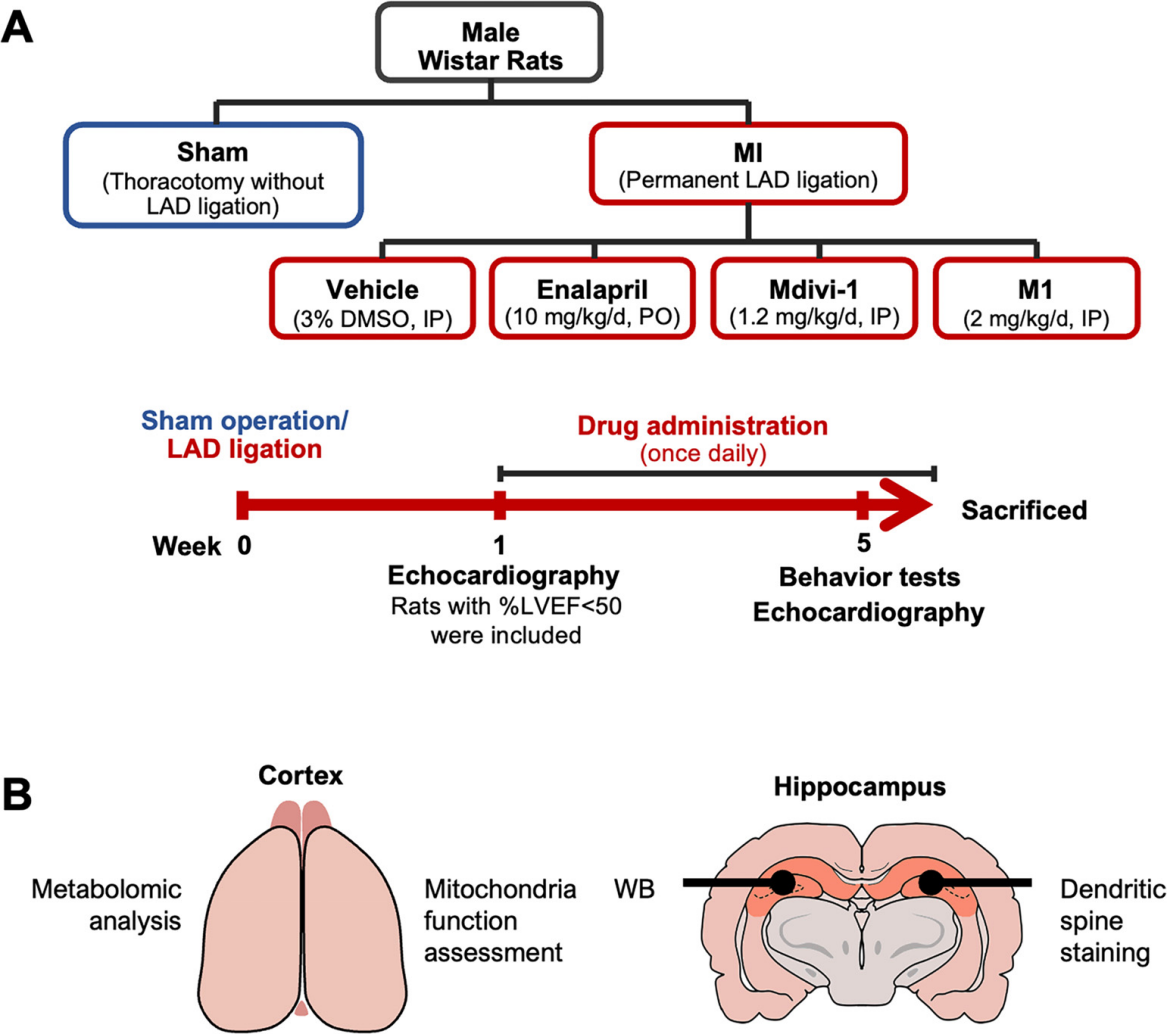
Schematic plan of the experimental protocol. (**A**) The experimental protocol of the study, (**B**) A diagram of brain sections for each experiment. **Abbreviations:** LAD: Left anterior descending; LVEF: Left ventricular ejection fraction; WB: Western blot; IP: Intraperitoneal injection; PO: Per oral.

## Data Availability

The data used in this study are available from the corresponding authors upon reasonable request.

## LIST OF ABBREVIATIONS

AD: Alzheimer’s Disease
Aβ: Amyloid Beta
LAD: Left Anterior Descending Coronary Artery
M1: Mitochondrial Fusion Promotor
Mdivi-1: Mitochondrial Division Inhibitor 1
MI: Myocardial Infarction
NOL: Novel Object Location Test
OFT: Open Field Test
OXPHOS: Oxidative Phosphorylation
ROS: Reactive Oxygen Species
