# Supplementary material for: Modulating Mitochondrial Dynamics Mitigates Cognitive Impairment in Rats with Myocardial Infarction
Source: Curr Neuropharmacol. 2024 Jan 31;22(10):1749–60. doi: 10.2174/1570159X22666240131114913 (PMC11284718; doi:10.2174/1570159X22666240131114913)
Supplement: Supplementary file 1 — Supplementary material is available on the publisher’s website along with the published article. [file CN-22-1749_SD1.pdf]

Supplementary Material

Modulating Mitochondrial Dynamics Mitigates Cognitive Impairment in Rats with Myocardial Infarction

Kewarin Jinawong[1,2,3], Chanon Piamsiri[1,2,3], Nattayaporn Apaijai[1,2,3], Chayodom Maneechote[1,2], Busarin Arunsak[1,2], Wichwara Nawara[1,2], Chanisa Thonusin[1,2,3], Hiranya Pintana[1,2], Nipon Chattipakorn[1,2,3] and Siriporn C. Chattipakorn[1,2,4,*]

[1]Neurophysiology Unit, Cardiac Electrophysiology Research and Training Center, Faculty of Medicine, Chiang Mai University, Chiang Mai 50200, Thailand; [2]Center of Excellence in Cardiac Electrophysiology Research, Chiang Mai University, Chiang Mai 50200, Thailand; [3]Cardiac Electrophysiology Unit, Department of Physiology, Faculty of Medicine, Chiang Mai University, Chiang Mai 50200, Thailand; [4]Department of Oral Biology and Diagnostic Sciences, Faculty of Dentistry, Chiang Mai University, Chiang Mai 50200, Thailand

1875-6190/24 $65.00+.00 **© 2024 Bentham Science Publishers**

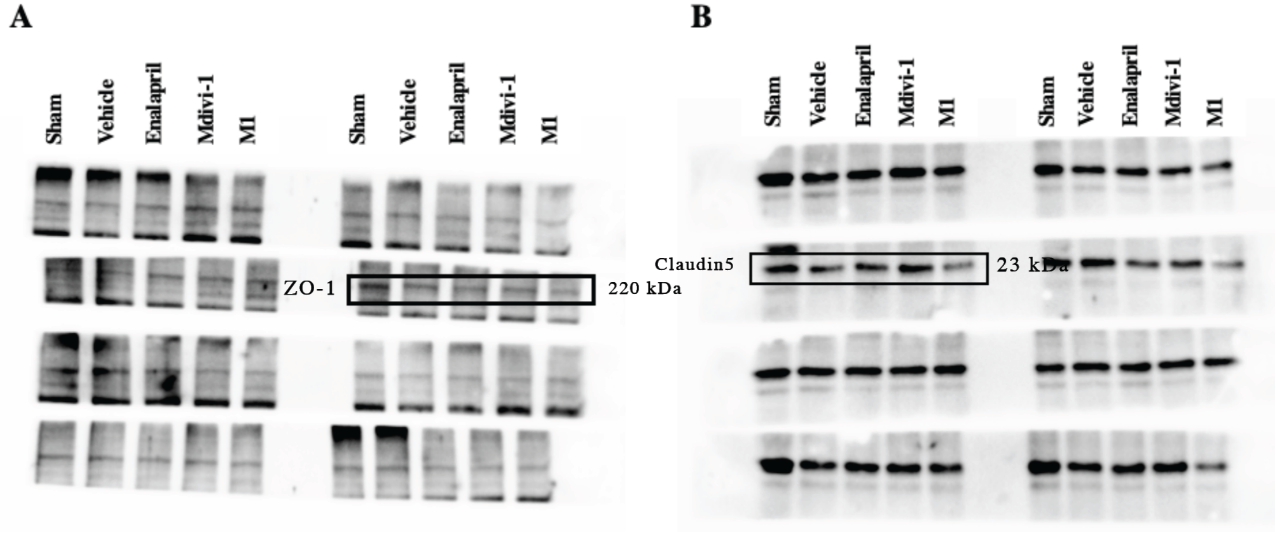

Fig. (S1). The original western blot of blood brain barrier protein expression. (**A**) The whole image of ZO-1 protein expression, (**B**) The whole image of claudin 5 protein expression.

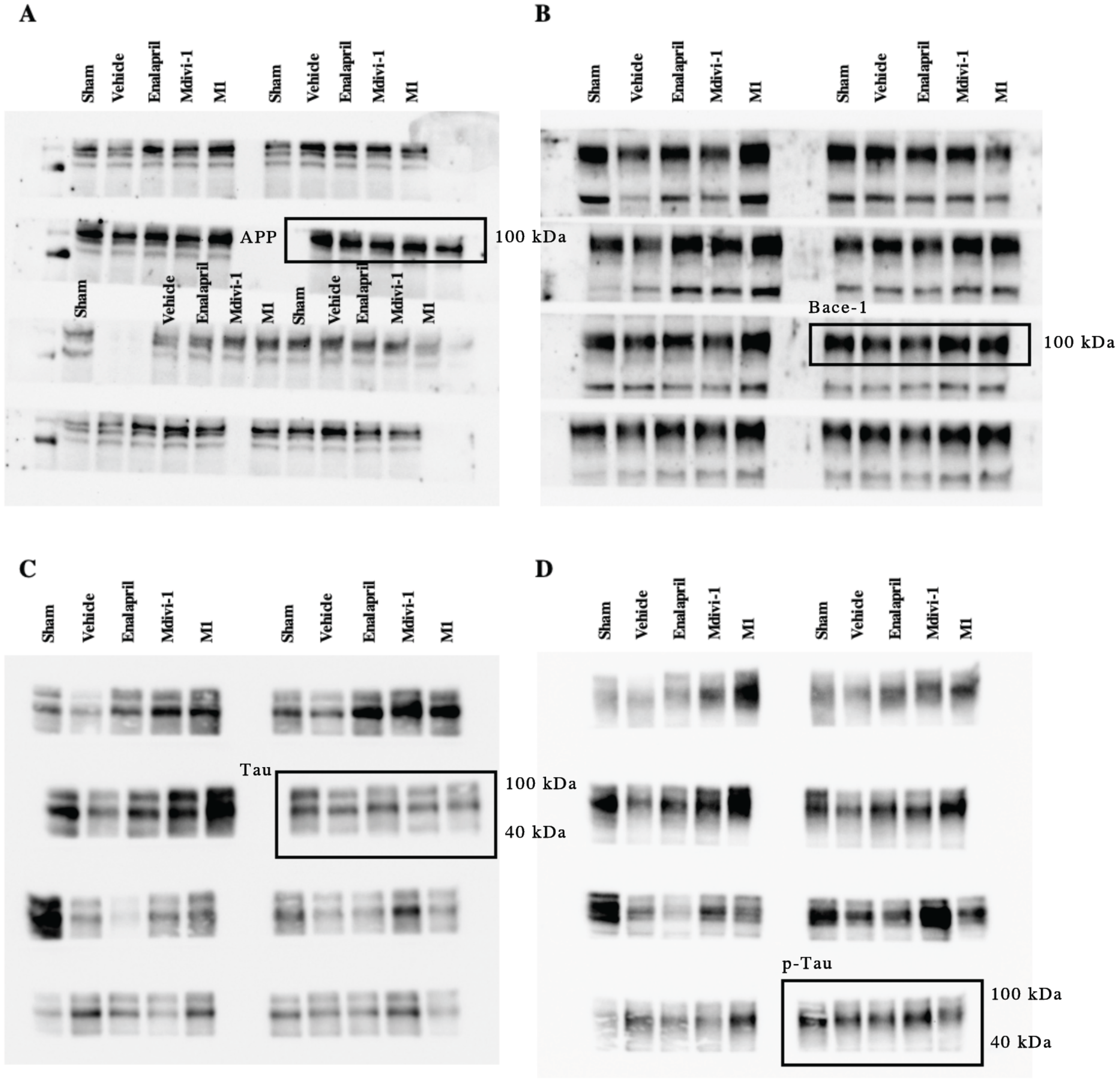

Fig. (S2). The original western blot of APP, Bace-1, Tau and p-Tau protein. (**A**) The whole image of APP protein expression, (**B**) The whole image of Bace-1 protein expression, (**C**) The whole image of Tau protein expression, (**D**) The whole image of p-Tau protein expression.

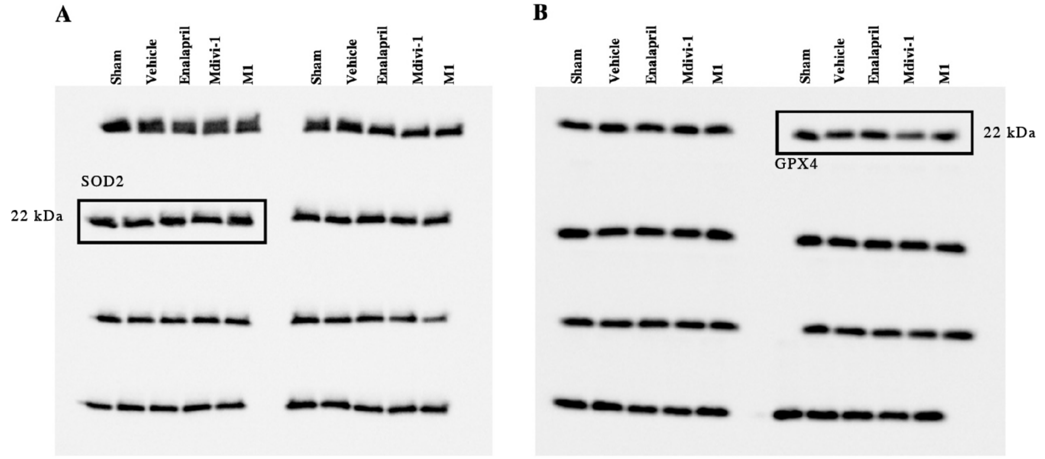

Fig. (S3). The original Western Blot of antioxidant protein expression. (**A**) The whole image of SOD2 protein expression, (**B**) The whole image of GPX4 protein expression.

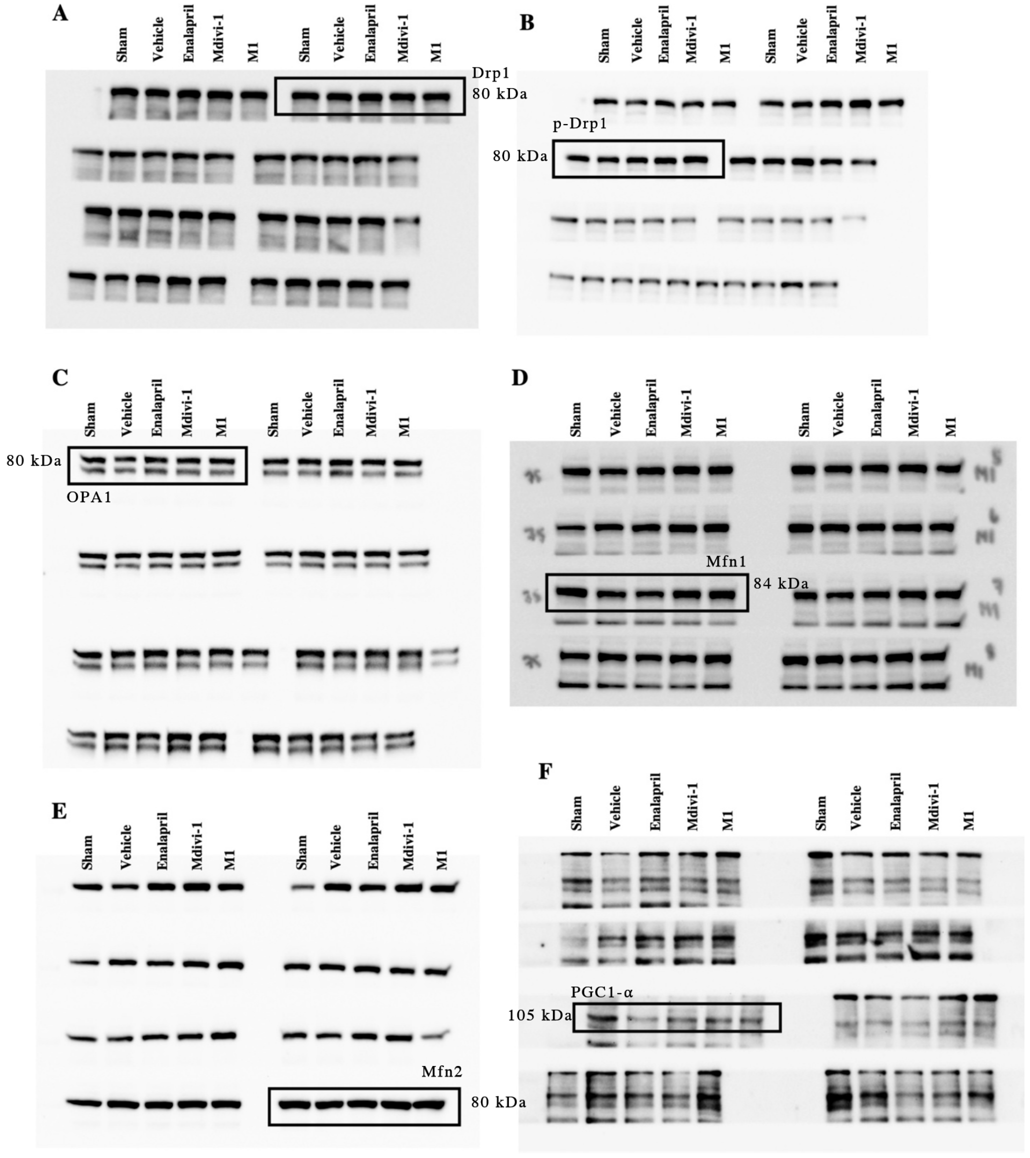

Fig. (S4). The original Western Blot of mitochondrial dynamics protein expression. (**A**) The whole image of Drp-1 protein expression, (**B**) The whole image of p-Drp1 protein expression, (**C**) The whole image of OPA1 protein expression, (**D**) The whole image of Mfn1 protein expression, (**E**) The whole image of Mfn2 protein expression, (**F**) The whole image of PGC1-α protein expression.

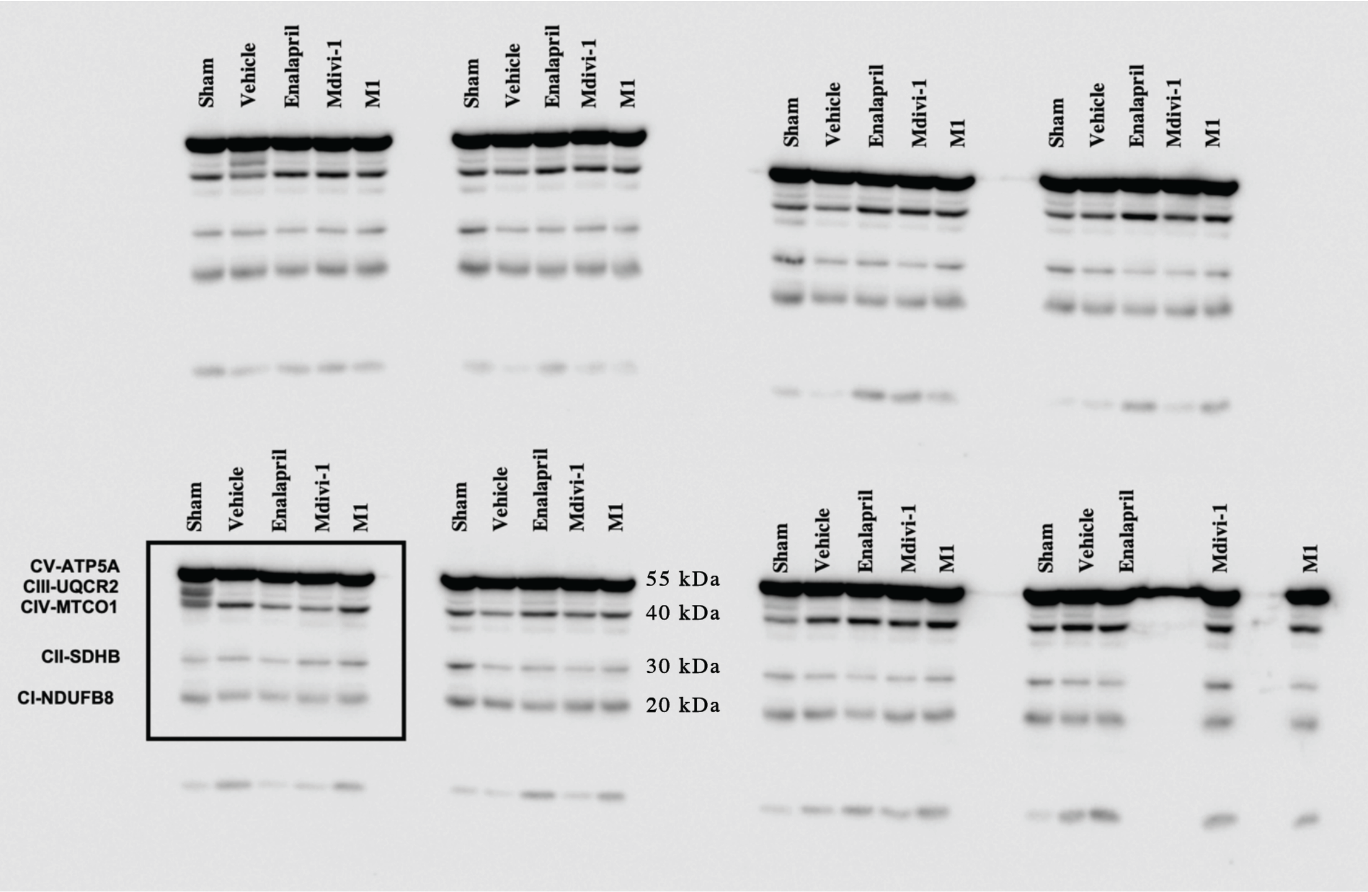

Fig. (S5). The original Western Blot of OXPHOS protein expression. The whole image of OXPHOS complex I-V expression.
